# The Role of Phosphatases in Nuclear Envelope Disassembly and Reassembly and Their Relevance to Pathologies

**DOI:** 10.3390/cells8070687

**Published:** 2019-07-07

**Authors:** Florentin Huguet, Shane Flynn, Paola Vagnarelli

**Affiliations:** College of Health and Life Science, Research Institute for Environment Health and Society, Brunel University London, London UB8 3PH, UK

**Keywords:** nuclear envelope (NE), nuclear pore complex (NPC), nuclear lamina, cyclin dependent kinase (CDK), polo-like kinase (PLK), protein phosphatase, micronuclei (MN)

## Abstract

The role of kinases in the regulation of cell cycle transitions is very well established, however, over the past decade, studies have identified the ever-growing importance of phosphatases in these processes. It is well-known that an intact or otherwise non-deformed nuclear envelope (NE) is essential for maintaining healthy cells and any deviation from this can result in pathological conditions. This review aims at assessing the current understanding of how phosphatases contribute to the remodelling of the nuclear envelope during its disassembling and reformation after cell division and how errors in this process may lead to the development of diseases.

## 1. Introduction

In higher eukaryotes, the chromosome condensation cycle, a necessary step for the faithful partitioning of the genetic material during cell division, is accompanied by a re-organization of the nuclear envelope (NE). At the onset of mitosis, the NE is disassembled, the entire genome is condensed into mitotic chromosomes, gene transcription and translation are attenuated and many DNA-bound proteins are removed from chromatin [1]. To reform a functional nucleus at the end of mitosis, all these modifications must be reversed and restored after division is accomplished [2]. These dramatic re-organization events are regulated by waves of phosphorylation, mainly as a result of kinase activity between mitosis onset and metaphase, and de-phosphorylation, as a result of phosphatase activity during mitotic exit.

Other reviews in this issue deal with the involvement of protein phosphatases in early mitotic events; here, we will focus on phospho-switches linked with the NE re-organization process during mitosis.

## 2. Nuclear Envelope Structure and Composition

One of the major characteristics of eukaryotes is the presence of a barrier, the NE, separating the contents of the nucleus from the cytoplasm. The NE is composed of an outer nuclear membrane (ONM), an inner nuclear membrane (INM)), nuclear pore complexes (NPC) and the nuclear lamina. ONM and INM extend from the membrane of the endoplasmic reticulum (ER), and similarly, the perinuclear space is a continuum with the ER lumen [3]. 

The NPCs are huge protein complexes (around 100–125 MDa [4]) spanning the NE that regulate restricted and bidirectional exchange of RNA and proteins between nucleoplasm and cytoplasm but also participate in genome organization and transcriptional regulation [5,6]. NPCs are composed of ~30 different nucleoporins (Nups) [7] organized in different subcomplexes: Y-complex, cytoplasmic complex, inner ring complex, transmembrane Nups, Nup62 complex and the nuclear basket complex [8].

In metazoan cells, the nuclear lamina associates with the nuclear side of the NE and is composed of lamins and fibrillar proteins (type V intermediate filaments) that form homodimers in head-to-tail conformation. Lamins belong to two different classes: A-type and B-type lamins. A-type lamins, which include Lamin A, Lamin AΔ10, Lamin C and Lamin C2, are coded by the *LMNA* gene, and B-type lamins, Lamin B1, Lamin B2, are coded by the *LMNB1* and *LMNB2* genes, respectively [9]. Several proteins, such as lamina-associated proteins 1/2 (LAP1/2), lamin B-receptor (LBR), emerin and others, associate with the nuclear lamina. The lamina not only provides elasticity and stiffness to the NE (attributed to A-type lamins), but the nuclear lamina is also involved in many cellular functions like chromatin tethering to the NE, chromatin organization, cytoskeleton linking, cell signalling and others [10]. 

These complex structures and protein interactions need to be re-organized to allow a successful mitosis. Therefore, an orderly dismantling of the NE occurs at the onset of cell division while a step-wise re-building takes place during mitotic exit. This is a tightly regulated process that also requires synchronization with the condensation/de-condensation cycle of the chromatin and other reorganizational processes of the cellular cytoskeleton.

## 3. Nuclear Envelope Disassembly

Nuclear Envelope Breakdown (NEBD) is a stage generally associated with the end of prophase and is directed by the phosphorylations of specific substrates by kinases such as cyclin-dependent kinase 1 (CDK1), Aurora kinases, Polo-like kinase (PLK) 1 and Never in mitosis gene a (NIMA)-related kinases. This process also requires the attenuation in the activity of the two main protein phosphatases, protein phosphatase 1 (PP1) and protein phosphatase 2A (PP2A) [11,12,13,14]. PP1, particularly the PP1 isoform PP1α, and PP2A activity have been shown to be inhibited by CDK-mediated phosphorylation. During mitotic entry, CDK1/CyclinB phosphorylates the C-terminus of PP1 [15]. At mitotic entry, PP2A-B55 is also inactivated [16]. This inactivation is possible as the Greatwall kinase (Gwl), activated by CDK1, phosphorylates ENSA/Arpp19 and allows it to bind PP2A-B55 leading to the inhibition of the phosphatase. This system also relies on the inactivation of PP1 via CDK1 phosphorylation [15,17,18].

In addition, there is also evidence to suggest that an interaction between the two phosphatases (PP1 and PP2A) is needed to ensure their inactivation in fission yeasts [18]. 

**Figure 1:**
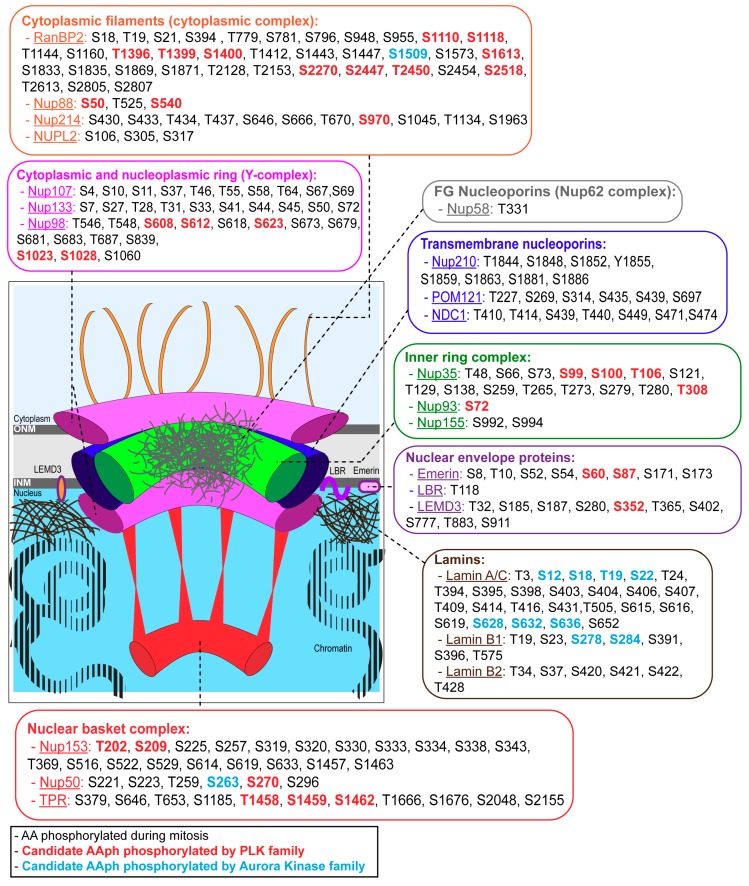
Nuclear envelope components phosphorylation during mitosis. A schematic representation of the nuclear pore complex (NPC) and its interactions with nuclear membranes (outer nuclear membrane (ONM) and inner nuclear membrane (INM)), lamins (in brown) and NE proteins (in purple). NPCs are composed by nucleoporins (NUPs) belonging to different subcomplexes: Y-complex (cytoplasmic and nuclear ring) (in pink), transmembrane NUPs complex (in blue), inner ring complex (in green), Nup62 complex (in grey), and nuclear basket complex (in red). During open mitosis, the nuclear envelope breaks down to allow chromosome segregation. This process is driven by phosphorylation. The mitosis-specific phosphorylated amino acids (AA) for each NUPs are listed based on published phosphoproteomic studies [21,22], together with the candidate kinases (PLK family in red and Aurora kinase family in blue) as identified in [23]. LBR: Lamin-B receptor; LEMD3: LEM domain-containing protein 3; NUPL2: Nucleoporin-like protein 2; POM121: Pore membrane protein 121kDa; RanBP2: Ran-binding protein 2.

The first step in NEBD and permeabilization of the nucleus is the disassembly of NPCs; this is mediated by the phosphorylation of Nups. Proteomic studies have identified a number of mitotic phosphosites in different Nups belonging to several subcomplexes (Figure 1) and indicated that Nup98 [19] and Nup35 [20] are heavily phosphorylated Nups in human cell mitosis. 

Several studies have started to unveil the molecular mechanism driving NPC disassembly at mitotic entry. Release of Nup98 (a component of the Y-complex) is one of the early events in NPC disassembly and occurs during prophase [24,25] due to a stepwise hyperphosphorylation of Nup98 by several mitotic kinases, including CDK1/Cyclin B and members of the NIMA-related kinase (NEK) family [19]. Recently, the role of PLK1 in promoting NPC disassembly has also been highlighted [20,26]. In agreement with the mitotic phosphoproteomic data suggesting that PLK1 is the highest represented kinase responsible for these phosphorylations (Figure 1), inhibition of PLK but not of Aurora B kinase leads to entry into mitosis with a complete Lamina A/C dispersal but an incomplete NPC disassembly [26]. The targets for PLK1 that appear to play a major role in the NPC breakdown are the scaffold nucleoporin Nup35 and the NPC gatekeeper Nup98. The initial phosphorylations in Nup98 occur in accessible and potentially unstructured regions. Phosphorylation of these regions could affect the overall configuration of the C-terminal domain of Nup98, perhaps by an allosteric mechanism which, in a concerted action with phosphorylations of other scaffold Nups, leads to the initial disassembly of the NPC. The role of the different phosphorylations identified in several Nups and their contribution to the final NPC disassembly are still to be determined.

Following this process, the phosphorylation of A-type lamins by CDK1/Cyclin B leads to the depolymerization of the nuclear lamina [27], while the disassembly of B-type lamins seems to be attributed to the activity of protein kinase C (PKC) in late prophase [28,29]. In vivo studies looking into the phosphorylation of B-type filaments identified that the Beta II isoform of PKC targets the S405 residue of Lamin B and facilitates its disassembly [30]. While lamins provide the structure to the INM, chromatin binding proteins, such as barrier-to-autointegration factor (BAF) (product of the BANF1 gene), mediate the association between chromatin and the NE. The phosphorylation of BAF by vaccinia-related kinase (VRK) 1 results in the dissociation of chromatin from the NE [31]. The consequence of a perturbed dissociation of nuclear membranes from chromatin during mitosis has been nicely illustrated in a recent work where a forced tethering was imposed between the NE and chromatin; in this condition, chromosome condensation defects and anaphase chromatin bridges were observed [32]. Disassembly of the nuclear lamina leads to the release of chromatin from NE membranes, and their retraction into the mitotic ER [33]. Therefore, from what we have discussed so far, it is clear that all these processes are possible due to the activation of CDK1 by the de-phosphorylation of its inhibitory Thr14 and Tyr15 residues at the onset of mitosis. These inhibitory phosphorylations are removed by Cdc25A and Cdc25B [34], thus, in an indirect manner, phosphatases are involved in NEBD and other major steps at the onset of mitosis. Before deciphering the mechanisms, it was already well known that inhibition of protein phosphatases could lead to premature chromosome condensation (PCC). Treatment of human peripheral lymphocytes using okadaic acid (OA) could cause PCC in a dose-dependent manner, irrespective of the cell cycle stage [35]. This technique has been used in several applications from radiobiology dosimetry [36] to clinical human karyotyping [37]. 

**Figure 2:**
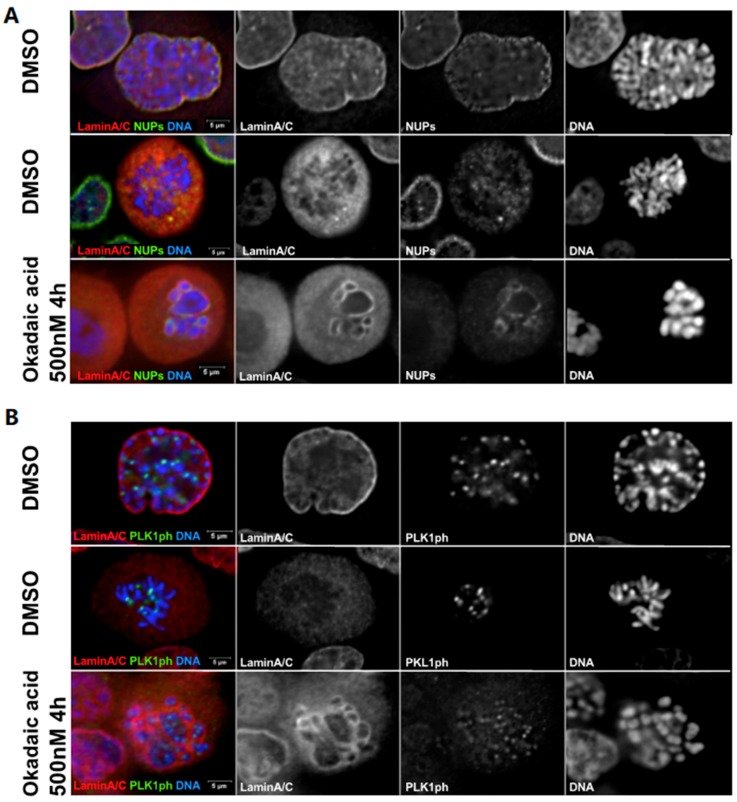
Okadaic acid triggers chromosome condensation but not complete NE break down. HeLa cells were treated with DMSO or 500 nM okadaic acid for 4 h, then fixed and stained for (**A**) Lamin A/C (red) and mAb44 (NPC components, green) or (**B**) phospho-PLK1 (T210) (green) and Lamin A/C (red). (**A**) In prophase, Lamin A/C and NUPs are not completely disassembled and chromosomes start condensing (top panel) while in prometaphase Lamin A/C and NUPs are completely disassembled and dispersed in the cytoplasm (middle panel). Upon okadaic acid treatment, chromosome condensation is as advanced as in prometaphase but Lamin A/C and NUPs are not completely disassembled and are still present around chromosomes (bottom panel). (**B**) In prophase, when Lamin A/C are still not disassembled, phosphorylated PLK1 (PLK1ph) already accumulates at the centromeres (top panel). In prometaphase, Lamin A/C is fully dispersed in cytoplasm and PLK1ph localizes to the centromeres (middle panel). After okadaic acid treatment, chromosomes are condensed similar to prometaphase, Lamin A/C are not completely disassembled and PLK1 starts accumulating at the centromeres (bottom panel).

Based on the current knowledge of cell cycle regulation and the kinases involved in the coordination between the dissolution of the NE and the condensation of chromatin (reviewed in [38,39]), it could be predicted that the induction of PCC would be triggered by the activation of CDK1, PLK1 (and its targeting to the centromere) and accompanied by Lamin A and NPC disassembly [26,27,28]. However, our unpublished work (Figure 2) does not suggest this to be the case. Induction of PCC by the inactivation of PP1 and PP2A by OA does indeed lead to PCC and targeting of phosphorylated (active) PLK1 to the centromere but it is not associated with the full dissolution of Lamin A and NPC in the majority of cases. Therefore, if the inactivation of PP1/PP2A is sufficient to drive chromosome condensation [35], it also suggests that other events need to occur to dismantle the NE and that the two processes can be uncoupled. This is one of the few examples whereby chromosome condensation and NE reorganization can be uncoupled during the cell cycle. The only other known case is provided by mutations in MCPH1 where chromosome de-condensation after mitosis is much delayed compared to the NE-reformation [40]. Clearly, this novel observation points to the need for further investigations on this process and on the regulation of the coordination between chromatin and nuclear membrane remodelling.

Once phosphorylated, some of the NE proteins accumulate in different regions of the mitotic cell, such as the mitotic spindle or kinetochores [41,42,43] and play roles in other mitotic events, such as chromosomal segregation [44]. Here we will not discuss the role of NE proteins in mitosis but [45,46,47] represent excellent reviews on this topic. In addition, during NE breakdown, the membrane component of the NE re-tracts into the ER which surrounds the mitotic spindle: this not only allows for the mitotic spindle to assemble but also facilitates the local concentration of mitotic factors that are essential for the chromosome–microtubule interaction [48].

## 4. Nuclear Envelope Reassembly

After the spindle assembly checkpoint (SAC) is satisfied, the anaphase-promoting complex (APC) is activated and triggers the degradation of cyclin B by the proteasome. Declining CDK levels is one of the requisites for mitotic exit together with the re-activation of PP1 and PP2A [49,50]. At this stage the NE starts reforming around the segregating chromatin. This reformation occurs in a sequential manner where the initial recruitment of Nups is followed by the assembly of the Lamina [51]. The role of phosphatases in this process is supported by many studies in several organisms.

### 4.1. Lamina Re-Assembly

In 2000, Steen et al. were the first to demonstrate that PP1 and its regulatory subunit A-kinase anchoring protein (AKAP) 149 were essential for NER in human cells [52]. PP1 and its regulatory subunit AKAP 149 is important for the localization of Lamin B to chromosomes during telophase, its dephosphorylation and consequent polymerization. This seems to be conserved in *Drosophila* where OA treatment prevents the accumulation of Lamin B-GFP around the segregating chromatin [53].

Lamin A is also heavily phosphorylated in mitosis (Figure 1) and these phosphorylations are removed during mitotic exit but candidate phosphatases for the reversal of these phosphosites are not yet known. However, it has been shown that de-phosphorylation of the S22 residue in Lamin A occurs via a transient recruitment of the protein to the chromatin in telophase where an interaction mediated by Sumo and a Sumo interacting motif (SIM) on Lamin A is essential for the process [54]. Interestingly, BAF, emerin, PP1 and Repo-man all contain a sumoylation site and therefore could potentially be involved in Lamin A de-phosphorylation. Recently, it has been shown that Arpp19 mouse knock out cells have a reduced level of Lamina S22 phosphorylation but there are no defects in the timing of Lamin A recruitment to the chromatin [55].

### 4.2. NPC Re-Assembly

Several studies have described NPC reconstruction as the timely association of Nups to chromatin, regulated by a mechanism involving Importin β and RanGTP [56]. Phosphoproteomic mapping of early mitotic exit events revealed that the de-phosphorylation of several Nups (107, 93 and 188) corresponded to the order of these proteins loading onto the chromatin and their recruitment to the reforming NPC, supporting the model of a dephosphorylation-driven timely reassembly [57]. Therefore, it could be argued that the dephosphorylation of NE proteins by the sequential activation of their corresponding phosphatase at specific times is important for correct NER.

One of the first steps involved in NPC re-assembly is the recruitment to the chromatin of ELYS (Embryonic Large molecule derived from Yolk Sac (AHCTF1))/MEL28 [58,59,60,61]. Besides being essential for NPC reformation, it has also been shown to be essential for recruiting PP1 in *Caenorhabditis elegans* [62]. In addition, ELYS/MEL28, is important for recruiting LBR to the chromosomal noncore region and for focusing of A-type lamin-binding proteins like emerin, Lap2α and the BAF at the chromosomal core region [63].

PP1 is also recruited to the segregating chromatin in anaphase by another targeting subunit: Repo-man [64]. This pathway is important for the recruitment of Importin β to the chromatin periphery during anaphase, a process negatively controlled by CDK phosphorylation of the Repo-man N terminal domain [2]; this mechanism is believed to be involved in the reassembly of NPCs. A non-phosphorylatable mutant of Repo-man that targets to the chromatin prematurely and binds stably to PP1, leads to a premature accumulation of some NPC components, namely Nup153 and Importin β but is not efficient in recruiting ELYS/MEL28 and Lamin A/C [2]. In this condition, the local activation of the phosphatase is sufficient to trigger some steps of NPC reformation even in the presence of high CDK1 and PLK1 activity [2]. Nup153 has been shown to be a critical component for the reformation of the nuclear pores in many systems [65]. Using a proteomic approach Cundell et al. showed that Nup153 (and RanBP2) is a candidate substrate for PP2A/B55 in anaphase and that its recruitment to the anaphase chromatin is delayed upon depletion of B55 [66] thus indicating that also PP2A plays a role in NE reformation. However, there are several mitotic phosphosites in Nup153 beside the ones that are affected by PP2A/B55 (see Figure 1) and the physical interaction between Nup153 and Repo-man/PP1 [67] raises the possibility that Nup153 is also a PP1 substrate.

PP2A has been shown to be responsible for the dephosphorylation of BAF [68]; during early mitosis, BAF phosphorylation by VRK1 suppresses its capability to bind DNA [31,69]. During mitotic exit, PP2A and its co-factor LEM4/ANKLE2 dephosphorylates BAF [68] to induce high-affinity DNA binding before membranes come in contact with chromatin. Its localization is also regulated by the geometry of NE membranes: once chromatin surface regions are covered by nuclear membranes, they cease to further accumulate as a consequence of nuclear membrane recruitment [70]. When the NE assembly is completed, BAF dissociates from chromatin [71]. The mitotic phosphoregulation of BAF, therefore, establishes a dense chromatin network in a small window of time during mitotic exit when nuclear membranes enwrap chromosomes [70]. It is interesting to note that, although not directly linked to mitosis, another phosphatase, PP4C, has been shown to dephosphorylate BAF [72]. In this study, the inhibition of BAF by RNAi, which essentially corresponds to altered phosphatase activity, exhibited characteristics mimicking those of progeroid syndrome, a subset of diseases usually attributed to the production of altered lamin proteins and abnormal NE. 

### 4.3. Membrane Re-Assembly

At the end of mitosis, NE membranes are also re-assembled around the segregated chromatin masses. The NE endoplasmic reticulum (ER) membranes are delivered to the rims of chromatin [73,74] and the resulting gaps are then sealed. Because the NE re-assembly process takes place when the anaphase spindle is still assembled, the spindle microtubules also need to be severed in order to allow the membrane fusion process to occur. These processes are mediated by the endosomal sorting complex required for transport (ESCRT) machinery [75,76,77,78]. The recruitment of the ESCRT complex to the telophase chromatin is mediated by CHMP7, a protein that interacts with ER membranes [78,79,80]. The C-terminal of CHMP7 (which is recruited by LEM2—Heh1 and Heh2 in yeast) on one hand triggers the polymerization of ESCRT-III components to seal the NE and, on the other, leads to the recruitment of Spastin, a microtubule-severing enzyme, to the same regions thus allowing for the coordination between spindle microtubule removal and membrane remodelling. Finally, VPS4 mediates the disassembly of ESCRT-III filaments and promotes the fusion of the NM, resulting in the complete sealing of the NE and the re-establishment of nuclear integrity and functionality [75,78,80]. See [81] for a review on the ESCRT III machinery and its membrane remodelling activity.

## 5. Potential for Disease

Numerous diseases ranging from cancer to progeria syndromes are characterized, at least in part, by deformities of the NE. 

Incorrect execution of mitosis results in a failure to produce healthy cells. In the presence of major defects, the cells will usually be eliminated by apoptosis, but small defects can also be extremely harmful. One example is represented by mistakes in chromosome segregation. Merotelic (kinetochore-microtubule) attachment is the main source of aneuploidy in human cells [82]; these chromosomes will dwell in the middle of the anaphase spindle and eventually incorporate into one of the two daughter cells resulting in the formation of an aneuploid cell. Although an increase/decrease in copy number could already represent a harmful situation, the major defect is actually linked to the dynamics of NE reformation. As we discussed earlier, NPC and nuclear membranes are recruited to the surface of the segregating chromatin in an orderly manner. In general, NPC components start assembling first in the poleward region of the chromatin and later on the central spindle side. Recent studies have shown that chromatin that is trapped within the central spindle fails in the assembly of several NPC components but it is capable of recruiting the Lamina [26,53,83]. This impairment in NE re-assembly will produce chromatin that is surrounded by the Lamina but lacks NPCs and is separated from the main nucleus of the cell. These structures, known as micronuclei (MN) are often found in cancer cells [84]. The negative impact that the formation of MN have on cells and their contribution to genome instability has been well demonstrated by several recent studies showing that these structures carry un-replicated DNA and DNA damage (the lack of NPCs causes an inability to sustain nuclear-cytoplasmic transport) that triggers chromothripsis, a chromosome shattering phenomenon that is at the basis of cancer evolution [83,84,85,86,87].

The complete molecular mechanisms are still in need of clarification, but two major hypotheses have been put forward. One is based on the fact that the central spindle is a region of high activity for Aurora B kinase and PLK1. Inhibition of Aurora B [26,53] and PLK1 [16] can trigger early assembly of the NPC. As it is known (Figure 1) that several Nups are phosphorylated by PLK1 and that their de-phosphorylation is essential for NPC re-assembly, the presence of the active kinase in the middle region of the chromatin could negatively regulate NPC re-assembly. 

The other hypothesis instead suggests that the spindle microtubules inhibit proper recruitment of these envelope proteins, independent of Aurora B [83], but the actual mechanism is less clear.

One consistent observation is that Nups are not loaded on chromatin that is trapped in the middle of the spindle and that de-phosphorylation of Nups must occur in order to promote their re-assembly [42,88,89]. In this respect, we cannot exclude that the key mechanism lies in the spatial activation of some yet unknown protein phosphatases. It could be possible that the key phosphatases are kept inactive in the vicinity of the spindle and via a kinase (maybe PLK1) thus preventing the local de-phosphorylation of key Nups. In this respect, it could recapitulate the situation present at the kinetochores in early mitosis, where a balance between kinase and phosphatase activities need to be achieved to move the process forward. 

Relative to diseases associated with the Lamina, Hutchinson-Gilford Progeria Syndrome (“HGPS” or “Progeria”), a premature ageing disease that resembles normal ageing in many key respects, is one of the most studied [90,91]. Progerin, a C-terminal mutant that lacks 50 amino acids of Lamin A and thereby retains a farnesyl group that is cleaved off in normal Lamin A, is the disease-causing defect in the majority of cases. It was recently shown that intact progerin is less responsive to dynamic mechanosensitive phosphorylations and degradation. Lamin A phosphorylation (S22), a major mitotic phosphosite, has been linked to increased Lamin A mobility and the production of a significantly softer nucleus [92]. 

Lamin A S22 has also been shown to be a target of CDK5. In neuronal cells and primary cortical neurons, CDK5 induces nuclear lamina dispersion by direct phosphorylation of lamin A and lamin B1. Phosphorylation-resistant mutants of lamins confer resistance to nuclear dispersion and cell death on neurotoxic stimulation, suggesting that this represents an early pathological event and a major mechanism for neuronal death [93]. In this respect, the level of phosphatase expression could also play a major role in the etiology of these diseases.

Interestingly, a case of heterozygous Lamin A S22L mutation has been reported in a patient suffering from dilated cardiomyopathy but no molecular data is available [94]. It would be interesting to evaluate how this mitotic phosphosite is involved in the pathogenesis and, since the mutation would produce a non-phosphorylable mutant, which phosphatase could be involved in cardiomyopathies. 

## 6. Conclusion and Future Perspective

We have recently witnessed a renaissance for protein phosphatases in many different fields including the study of the cell cycle. These critical phase transitions and the cell choices are, in the end, made on the balance between the activities of kinases and their counteracting phosphatases. Great examples have been provided by mitotic entry, the establishment and removal of the SAC and mitotic exit execution. A great level of molecular details have been acquired for the checkpoint regulation [39] but for a mitotic exit, the road is still quite bumpy mainly due to the need of new tools to investigate the role of specific phosphatases in this rapid and dynamic phase. However, the availability of systems for rapid protein degradation, optogenetic tools and single cell analyses will provide the right platform to carry out more investigations that will lead to the identification of substrates, their sequential de-phosphorylation and acquisition of knowledge on the different phosphatase specificities.
